# Application of Semiconductor Metal Oxide in Chemiresistive Methane Gas Sensor: Recent Developments and Future Perspectives

**DOI:** 10.3390/molecules28186710

**Published:** 2023-09-20

**Authors:** Li Fu, Shixi You, Guangjun Li, Xingxing Li, Zengchang Fan

**Affiliations:** 1 Key Laboratory of Novel Materials for Sensor of Zhejiang Province, College of Materials and Environmental Engineering, Hangzhou Dianzi University, Hangzhou 310018, China; lixingxing@hdu.edu.cn; 2 Research and Development Center, Siterwell Electronics Co., Ltd., Ningbo 315000, China; charlie@china-siter.com (G.L.); frank@china-siter.com (Z.F.)

**Keywords:** semiconductor metal oxide, chemiresistive methane gas sensor, sensitivity, selectivity, sensor fabrication, material science, machine learning, green manufacturing

## Abstract

The application of semiconductor metal oxides in chemiresistive methane gas sensors has seen significant progress in recent years, driven by their promising sensitivity, miniaturization potential, and cost-effectiveness. This paper presents a comprehensive review of recent developments and future perspectives in this field. The main findings highlight the advancements in material science, sensor fabrication techniques, and integration methods that have led to enhanced methane-sensing capabilities. Notably, the incorporation of noble metal dopants, nanostructuring, and hybrid materials has significantly improved sensitivity and selectivity. Furthermore, innovative sensor fabrication techniques, such as thin-film deposition and screen printing, have enabled cost-effective and scalable production. The challenges and limitations facing metal oxide-based methane sensors were identified, including issues with sensitivity, selectivity, operating temperature, long-term stability, and response times. To address these challenges, advanced material science techniques were explored, leading to novel metal oxide materials with unique properties. Design improvements, such as integrated heating elements for precise temperature control, were investigated to enhance sensor stability. Additionally, data processing algorithms and machine learning methods were employed to improve selectivity and mitigate baseline drift. The recent developments in semiconductor metal oxide-based chemiresistive methane gas sensors show promising potential for practical applications. The improvements in sensitivity, selectivity, and stability achieved through material innovations and design modifications pave the way for real-world deployment. The integration of machine learning and data processing techniques further enhances the reliability and accuracy of methane detection. However, challenges remain, and future research should focus on overcoming the limitations to fully unlock the capabilities of these sensors. Green manufacturing practices should also be explored to align with increasing environmental consciousness. Overall, the advances in this field open up new opportunities for efficient methane monitoring, leak prevention, and environmental protection.

## 1. Introduction

The surge in industrialization and technological progress has introduced a myriad of challenges pertaining to environmental monitoring, within which gas sensing plays a pivotal role [1]. As a crucial component of environmental safety and health management systems, gas-sensing technology is deployed to discern and track the concentration of diverse gases in the atmosphere [2]. Of these, the detection of methane, a potent greenhouse gas, assumes substantial importance due to its environmental and safety ramifications.

Methane, the principal component of natural gas, holds a critical role in both environmental and industrial spheres [3]. On the environmental front, methane is a primary catalyst for global warming, possessing a global warming potential twenty-five times greater than carbon dioxide over a century [4]. Hence, expedient detection and quantification of methane emissions are indispensable to climate change mitigation strategies [5]. Conversely, in industries such as petroleum, natural gas, mining, and waste management, the unregulated discharge of methane can give rise to perilous conditions owing to its high flammability and explosiveness [6]. Consequently, the adoption of sophisticated methane-sensing technologies can make a substantial contribution to both environmental preservation and industrial safety [7]. Table 1 shows examples of industrial methane sensors and maximum detection ranges.

Against this backdrop, chemiresistive gas sensors have drawn considerable interest due to their uncomplicated structure, cost-effectiveness, and facile integration with electronic devices [8]. These sensors operate on a straightforward yet efficient principle: the resistance of the sensing material undergoes a change in the presence of the target gas. Gas molecules interact with the sensing material, modifying its conductance or resistance, which is subsequently measured to ascertain the gas concentration [9]. Table 2 shows the comparison of the chemiresistive sensors with other types of methane sensors. Chemiresistive sensors offer numerous benefits such as cost-efficiency, compactness, low power requirements, and real-time detection, but they are not devoid of their shortcomings. One of the main challenges associated with chemiresistive sensors lies in the issue of selectivity [10], that is, their response to gases other than the target gas. Moreover, these sensors frequently necessitate high operating temperatures, which can culminate in increased power consumption and restrict their use in specific environments [11]. Lastly, the long-term stability of chemiresistive sensors remains a pivotal challenge that necessitates resolution [12].

Addressing these challenges and enhancing the performance of chemiresistive sensors necessitate the introduction of innovative materials, and in this regard, metal oxide semiconductors have emerged as promising candidates. These materials showcase a unique blend of properties such as high surface reactivity, tunable electrical properties, and environmental resilience, positioning them ideally for deployment in chemiresistive sensors [8]. Metal oxides such as zinc oxide (ZnO), tin oxide (SnO_2_), and titanium dioxide (TiO_2_) have been rigorously researched and utilized in the fabrication of chemiresistive gas sensors.

Moreover, the choice of the metal oxide semiconductor significantly impacts the sensor’s sensitivity, selectivity, response and recovery times, and stability. For example, SnO_2_-based sensors are renowned for their high sensitivity and swift response times [13], while TiO_2_-based sensors exhibit excellent selectivity [14]. Recent advancements in nanostructuring, hybrid materials, and other sophisticated techniques have further amplified the capabilities of these metal oxide semiconductors, paving the way for highly sensitive and selective methane sensing [15,16,17].

This review paper provides a comprehensive examination of the application of semiconductor metal oxides in chemiresistive methane gas sensors. It delves into the foundational mechanisms and operational principles of these sensors, underscoring their potential for versatile and efficient gas detection. The paper presents an overview of the types of metal oxide semiconductors commonly employed, their corresponding performance characteristics, and the recent advancements in enhancing their sensitivity, selectivity, response and recovery times, and long-term stability. Additionally, it thoroughly investigates the challenges and limitations associated with these sensors. Future research trajectories, encompassing material innovations, sensor design enhancements, and data analysis techniques, are explored to guide the development of more effective and reliable methane detection solutions.

## 2. Chemiresistive Gas Sensors: Fundamentals and Mechanisms

Chemiresistive gas sensors have become the focus of substantial research due to their simplistic design, user-friendly nature, and versatile application potential. The fundamental operational principle of these sensors is predicated on a change in electrical resistance induced by a chemical reaction [18]. Essentially, chemiresistive sensors convert a chemical signal, represented by the presence or absence of a target gas molecule, into an electrical signal that can be measured and quantified [19].

The working mechanism of chemiresistive sensors depends on the interaction between the target gas molecules and the sensing material. Upon exposure to a gas, the molecules physically adsorb onto the surface of the sensing material, instigating a chemical reaction [20]. This reaction leads to a transfer of charge carriers between the target gas concentration and the sensing material, consequently altering the material’s resistance. The change in resistance is directly proportional to the concentration of the target gas, thereby enabling its detection and quantification.

**Figure 1:**
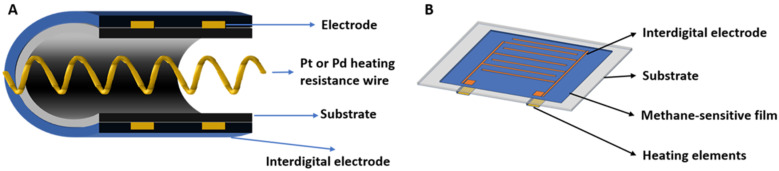
Schematics of (**A**) sintered-type and (**B**) thin-film-type chemiresistive methane gas sensors.

Semiconductor chemiresistive sensors, depending on their structural form, can be further categorized into sintered, thick-film, thin-film, multilayer film, and silicon microstructure variants [21]. The architecture of a fundamental chemiresistive sensor encompasses four key components: the sensing layer, electrodes, a heating electrode, and a substrate [22]. The sensing layer, constituted by a chemiresponsive material, is the most vital element as it directly engages with the target gas. The electrodes, typically fabricated from conductive materials like gold or platinum, are utilized to measure the resistance change in the sensing material [23]. These components are assembled on a substrate, commonly made from an insulating material such as alumina, which imparts physical support to the sensor. As illustrated in Figure 1A, the sensor under discussion is a sintered-type, side-heated model, typically utilizing a ceramic tube as its substrate on which electrode leads are soldered. The ceramic tube interior accommodates a heating resistance wire, and subsequently, a slurry, prepared from a blend of methane-sensitive material and a small quantity of adhesive, is uniformly applied to the substrate. The manufacturing technique of this sensor is simple and cost-effective, albeit unsuitable for large-scale production. Thin-film sensors, as depicted in Figure 1B, employ methods such as chemical vapor deposition, vacuum sputtering, and sol–gel processes to deposit a layer of methane-sensitive thin film on ceramic substrates pre-equipped with electrodes and heating elements. Although the fabrication process of thin-film gas sensors is intricate and cost-intensive, it facilitates lower consumption of sensitive materials, a larger specific surface area, and superior repeatability and mechanical strength of the sensor.

The sensing layer, regarded as the core of a chemiresistive sensor, profoundly influences the sensor’s performance due to its inherent properties. This sensing material necessitates a high affinity for target gas molecules to effectively adsorb them and incite a significant change in resistance [24]. In addition, the material should possess favorable electrical properties, facilitating a distinct and measurable change in resistance. A wide variety of methane-sensitive materials exist [25]. Upon examining the principles that govern interactions between these materials and methane gas molecules, methane-sensitive substances can be classified into four categories: semiconductors and their composite materials, composite materials of organic polymers, supramolecular host–guest systems A, and carbon nanotubes (CNT) along with their doped materials [26]. Among these, semiconductor materials have emerged as an optimal choice for the sensing layer in chemiresistive sensors. Semiconductors are materials that exhibit electrical conductivity intermediate between that of conductors (like metals) and insulators (like glass). The electrical properties of semiconductors can be readily adjusted by altering their temperature, introducing impurities, or modifying their structure, making them ideally suited for sensing applications.

Metal oxide semiconductors, notably, have demonstrated high effectiveness in chemiresistive gas sensors due to their unique properties that are aptly suited for this role. To start with, metal oxides, particularly in nanostructured forms, exhibit a high surface-to-volume ratio, thereby offering an expansive surface area for gas adsorption [26]. Secondly, their high surface reactivity augments their interaction with gas molecules. Lastly, metal oxides exhibit significant changes in electrical resistance upon exposure to different gases, enabling high sensitivity in gas detection [27]. Semiconductor metal oxides used for methane detection typically fall into two categories: n-type and p-type semiconductor oxides. Within this framework, n-type semiconductor oxide materials predominantly utilize free electrons as charge carriers, thereby experiencing a resistance decrease upon interaction with methane gas. In contrast, p-type semiconductor oxide materials primarily employ holes as the main charge carriers, leading to a resistance increase in the presence of methane gas [28]. Given the extensive use of SnO_2_ as a methane-sensitive metal oxide semiconductor material, it will serve as a representative of n-type semiconductor oxides to elaborate on the gas sensitivity mechanism between semiconductor metal oxides and methane gas. Currently, several theoretical models elucidate the gas sensitivity mechanism of SnO_2_ gas-sensitive films, including the surface space charge model, grain boundary potential barrier model, adsorption effect model, and adsorbed oxygen model [29,30,31,32]. Among them, the adsorbed oxygen model was the most commonly used explanation [32].

**Figure 2:**
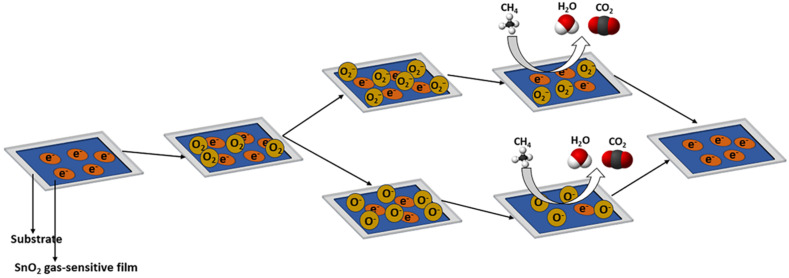
Schematics of model of oxygen adsorption–desorption sensing mechanism for methane gas detection.

The adsorbed oxygen model is currently the most widely acknowledged theoretical explanation for the gas sensitivity mechanism [32]. As depicted in Figure 2, the polycrystalline SnO_2_ semiconductor is distinguished by its numerous grains and grain boundaries. In comparison to monocrystalline materials, polycrystalline materials generate local potential barriers between grains. The film’s surface and interparticle interface electrical properties are influenced by the adsorption and desorption of gas molecules. When exposed to air, the resistive semiconductor sensor initially physically adsorbs oxygen molecules from the air onto the surface of the SnO_2_ gas-sensitive film. As the temperature rises, these physically adsorbed oxygen molecules absorb activation energy from the semiconductor surface and capture electrons, thus forming O_2_^−^, chemically adsorbed oxygen ions. These oxygen molecules attract electrons from SnO_2_’s conduction band, thereby increasing the sensor’s resistance. Under higher temperatures, the O_2_^−^ anion attracts an additional electron to form the O^−^ anion. At this stage, when methane gas encounters the resistive SnO_2_ gas-sensitive film, a chemical reaction occurs between the adsorbed oxygen and the methane gas due to the reductive properties of the latter. This reaction releases electrons back into the SnO_2_ conduction band, leading to an increased electron concentration in the conduction band and a subsequent decrease in resistance on the n-type semiconductor’s surface.

Nanocrystalline refers to a material, whether single or multi-phase, whose dimensions have been reduced to between 1 and 100 nm on at least one axis. As these crystals decrease in size, more of their surface area is exposed, resulting in a higher proportion of atoms at the grain boundary. This boundary houses defects such as vacancies and dangling bonds, which notably impact electron transportation properties. Xu et al. [33] proposed a model elucidating the relationship between the oxygen adsorption-induced depletion layer, crystal size, and the heightened response of nanocrystalline metal oxide gas sensors. Following this, Rothschild and Komen [34] exhibited a linear increase in conductivity as trapped charge densities decrease, and they showed that the reaction to gas-induced trapped charge density fluctuations inversely correlates with the average grain size.

**Figure 3:**
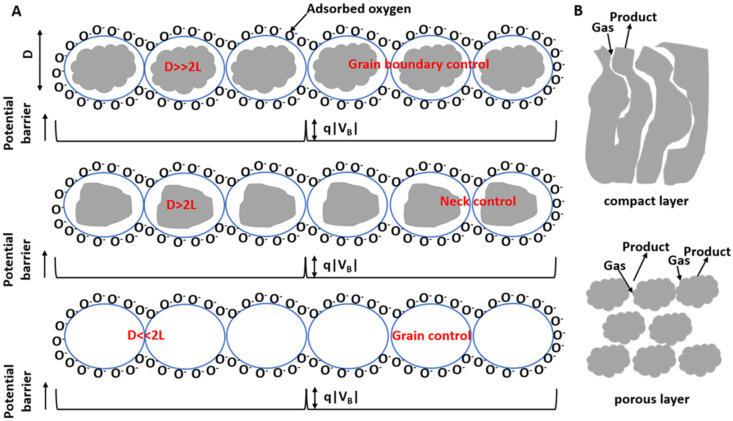
(**A**) Schematic of nanocrystalline ZnO thin films and the space charge region around the surface of each grain at inter-grain contacts. (**B**) Schematic view of gas-sensing reaction in compact layer and porous layer.

Figure 3A visualizes several grains of nanocrystalline metal oxide thin films and the space charge region surrounding each grain’s surface at the inter-grain junctions. Devoid of electrons, this region presents a higher resistance than the bulk. When these sensors encounter reducing gases, the electrons held by the adsorbed oxygen return to the oxide grains, decreasing both the potential barrier height and resistance. The individual crystals in these gas-sensing units are connected to neighboring crystals through grain boundary contacts or necks. It was observed that a heightened response arises when the grain size is notably less than double the depletion width [35]. In such instances, the depletion region extends throughout all grains, nearly depleting them of electrons. This results in reduced conductivity at the junction, causing a significant change in conductivity in the presence of reducing gases and a high response.

Moreover, nanocrystalline metal oxides can decrease the operating temperature of gas sensors. Zhang et al. [36] discovered that surface or interfacial tension declines as particle size reduces due to a potential energy increase in the particles’ bulk atoms. Smaller particles, with heightened molar free energy, exhibit a stronger tendency for adsorption of molecules or ions per unit area onto their surfaces, decreasing the total free energy and improving stability. As a result, smaller particles have a higher adsorption coefficient for gases, facilitating the adsorption of oxygen or reducing gases onto the nanocrystalline metal oxide surface with relative ease.

In the case of solid metal-oxide-sensing layers, gas permeation is obstructed, confining gas-sensing reactions strictly to the surface. However, porous layers allow gases to permeate throughout the entire sensing layer, facilitating gas-sensing reactions not only on the surfaces of individual grains but also at grain boundaries and grain–electrode interfaces, as illustrated in Figure 3B. Consequently, porous layers have demonstrated superior efficacy in methane detection when compared to compact layers, as documented in previous studies [33,37,38,39].

The thickness of the metal oxide thin films plays a substantial role in sensor responses. To achieve optimal responses from metal-oxide-based gas sensors, the thickness of the electron-depleted region should closely correspond to the thickness of the metal oxide thin films. It is generally observed that sensors constructed from thinner films tend to exhibit a significantly higher response than their resistive counterparts [40,41].

A correlation between the steady-state response of the sensor and the thickness of the sensitive film employed was proposed by Babaei and Orvatinia [42]. Their findings indicated that the response decreases exponentially as the thickness of the sensitive film augments. Interestingly, multiple researchers have reported that the gas response of sensors can be amplified with certain combinations of structural parameters, such as porosity and the presence of cracks [39,43].

The selection of the metal oxide semiconductor is instrumental in defining the sensor’s sensitivity, selectivity, and response times. For instance, SnO_2_ is commonly employed owing to its high sensitivity toward reducing gases. Waitz et al. [44] proficiently synthesized mesoporous SnO_2_ gas sensors possessing high thermal stability via nanocasting from ordered mesoporous KIT-6 silica. The gas-sensing properties of these mesoporous SnO_2_ sensors were evaluated and demonstrated encouraging responses to methane at elevated operating temperatures of 500 and 600 °C. The sensors exhibited prompt response times, approximately 1 to 2 min, and their response to methane concentrations showed a near-linear relationship at these temperatures. Furthermore, semiconductors such as ZnO, VOx, Co_3_O_4_, In_2_O_3_, and WO_3_ are routinely employed in methane-sensitive gas sensors. Each material has its own advantages and limitations based on its electronic, chemical, and structural properties. For instance, ZnO with its wide bandgap and high electron mobility shows good sensitivity to methane at moderate temperatures [45] while VO_2_ operates at lower temperatures [46]. Co_3_O_4_ provides a highly stable and selective response due to its p-type conductivity and suitability for high-temperature operation [47]. In_2_O_3_ offers low power consumption and fast response times but has limitations in selectivity [48]. WO_3_ is also stable and selective but can demonstrate slower response times [49].

The function of semiconductor materials in chemiresistive sensors extends beyond their role as the sensing layer. They also facilitate the enhancement of the sensor’s overall performance through various modification strategies, discussed in detail in Section 4. For instance, doping the semiconductor with different elements can bolster the sensor’s sensitivity and selectivity. In a similar vein, modifying the morphology of the semiconductor, such as generating nanoporous structures, can augment the surface area available for gas adsorption, thereby optimizing the sensor’s response time.

## 3. Types of Metal Oxide Semiconductors for Methane Sensing

Chemiresistive sensors have garnered substantial interest in the field of methane detection due to their distinctive capability to convert chemical data into easily interpretable electronic signals. Among the broad spectrum of materials suitable for chemiresistive sensors, metal oxide semiconductors have emerged as particularly proficient in methane sensing [50]. Various types of metal oxide semiconductors have been investigated for this application, each demonstrating a unique set of performance characteristics, along with their respective advantages and disadvantages.

SnO_2_ ranks as one of the most extensively studied and commonly used metal oxide semiconductors in gas sensing. Its high sensitivity toward a multitude of reducing gases, including methane, is notable. The high responsiveness of SnO_2_ to methane is largely attributed to its broad bandgap and superior electron mobility. For example, Xue et al. [51] reported porous 3D SnO_2_ nanomaterials for methane sensing. The gas-sensing properties of the sensor exhibited a lower initial response temperature (80 °C) and optimal working temperature (120 °C) for methane detection. The sensor also demonstrated better linearity in the methane concentration range (100–3000 ppm), excellent stability, and better selectivity to methane.

ZnO is another frequently selected metal oxide semiconductor for methane sensing. Its broad bandgap and remarkable electron mobility contribute significantly to its high gas-sensing performance [52]. Moreover, ZnO demonstrates excellent thermal stability, rendering it appropriate for applications demanding high operating temperatures. For example, Zhou et al. [45] synthesized various hierarchical ZnO nanostructures (flower-like nanorods, net-like nanofibers, and nanobulks) using a hydrothermal method for gas-sensing applications, specifically methane detection in transformer oil due to faults. The main findings revealed that the sensors based on hierarchical ZnO nanorods and nanofibers exhibited superior performance compared to nanobulks.

Although not as widely utilized as SnO_2_ and ZnO, TiO_2_ exhibits potential for methane detection. The heightened reactivity of TiO_2_’s surface, combined with its capacity to operate at reduced temperatures, renders it an appealing candidate for methane sensors. For example, Comert et al. [53] fabricated a methane sensor using TiO_2_ thin films at low operating temperatures. The TiO_2_ thin films were deposited on n-Si substrate using radio frequency (RF) magnetron sputtering and annealed at different temperatures. The gas sensor operated at 50 °C showed sensitivity to methane gas, and its detection performance increased with higher temperatures. The sensor’s average sensitivity per ppm was 0.15, and it showed a linear response in the concentration range of 100–1000 ppm methane gas.

WO_3_ is known to demonstrate substantial sensitivity toward methane. Its elevated surface reactivity and broad bandgap enhance its sensing performance. Furthermore, WO_3_ has been identified as having superior selectivity toward methane in comparison to certain other metal oxides. For example, Stankova et al. [49] investigated the gas-sensing properties of WO_3_ thin films deposited by RF sputtering on silicon substrates. Notably, the study reported a high sensitivity of the WO_3_ films toward methane. The gas-sensing activity of different surfaces of hexagonal WO_3_ in relation to methane activation and sensing was explored using density functional theory (DFT) calculations [54]. The main findings indicate that the flat (001) surface displayed low activity for methane sensing, with neither the oxygen-poor WO-terminated nor the oxygen-rich O-terminated surface effectively interacting with methane. However, the ridge-like (110) surface demonstrated highly active behavior, enabling efficient methane activation and sensing. On this surface, significant charge transfers occurred on active sites, such as W4c and hole2, and the methane molecule could approach as close as 2.317 Å to the surface, leading to a fourfold improvement in sensing ability compared to the (001) surface.

Other types of metal oxide semiconductors that can be used for methane detection include Co_3_O_4_, NiO, In_2_O_3_ [55], and VO_2_. For example, Shaalan et al. [47] synthesized Co_3_O_4_NPs using microwave irradiation and investigated their gas-sensing properties, particularly for methane detection. The gas sensor based on Co_3_O_4_NPs showed promising results, with a faster response and recovery time at low temperatures. Specifically, at an operating temperature of 200 °C and a methane concentration of 1%, the response time was 100 s, and the recovery time was 50 s. The sensitivity of the sensor improved with higher operating temperatures and gas concentrations. The study demonstrated that Co_3_O_4_NPs have potential as an effective sensing material for methane gas detection, which is essential for safety control and preventing hazards in gas systems. Prasad et al. [56] investigated the gas-sensing properties of newly developed VO_2_ nanostructured films for detecting methane near room temperature. The researchers synthesized single-phase VO_2_ nanostructures using pulsed dc-magnetron sputtering and controlled oxidation. Gas-sensing experiments revealed that the VO_2_ films responded well to methane at temperatures as low as 50 °C, showing a maximum response of 1.4% for 50 ppm methane at this temperature. Wang et al. [48] recently investigated the gas-sensing performance of Ag-modified In_2_O_3_ microspheres for methane detection. They synthesized different Ag/In_2_O_3_ composites with varying Ag contents and tested their sensing properties. The results showed that the 6%-Ag/In_2_O_3_ composite sensor exhibited the highest response to methane at 120 °C, which was about two times higher than the pure In_2_O_3_ sensor. It also had a lower detection limit of 0.7 ppm and excellent selectivity for methane over other interfering gases commonly found in coal mines.

Key performance characteristics that delineate the efficacy of a gas sensor encompass sensitivity, selectivity, response and recovery time, and stability. Sensitivity is typically highest toward methane in SnO_2_ among the metal oxides discussed due to its wide bandgap and exceptional electron mobility, which together enable a substantial change in resistance upon gas adsorption. ZnO and WO_3_ also display commendable sensitivity, while TiO_2_ generally exhibits reduced sensitivity, particularly at ambient temperature.

**Figure 4:**
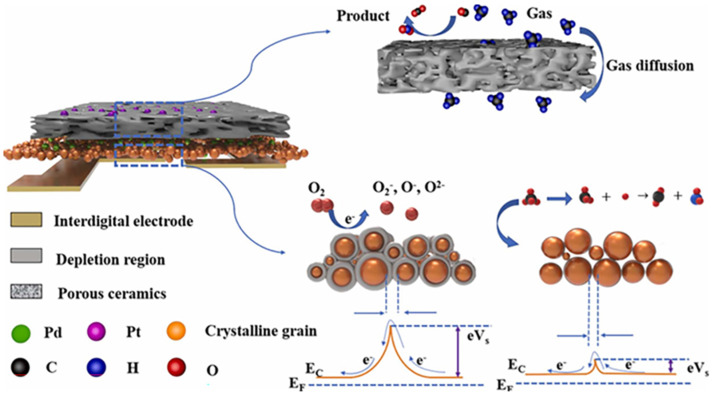
The schematic drawing of sensing mechanism for sensors equipped with an on-chip microfilter exposed to air and methane. Reproduced with permission from Ref. [57].

Selectivity is notably exhibited by WO_3_, which demonstrates superior selectivity toward methane over other gases. SnO_2_ and ZnO, although sensitive to methane, often display significant responses to additional gases, which can impede precise methane detection. While TiO_2_ exhibits acceptable selectivity, it is frequently overshadowed by the superior performance of WO_3_. Wu et al. [57] introduced a novel sensor structure with an on-chip microfilter made of porous ceramic impregnated with Pt to enhance methane detection selectivity and reduce interference from other gases. The microfilter acts as a catalyst, effectively converting interfering gases like CO and ethanol into inactive species, thereby preventing them from interfering with methane detection (Figure 4). The results showed that traditional metal oxide semiconductor sensors exhibited significant cross-sensitivity to CO and ethanol when exposed to methane, potentially leading to inaccurate readings. However, with the on-chip microfilter, the sensor’s response to methane was greatly improved, and it selectively detected methane even in the presence of interfering gases like CO and ethanol. The microfilter effectively filtered out these interferences, maintaining the underlying sensing mechanism of the sensor, which relies on surface-adsorbed oxygen species. The study focused on developing a technique for the selective detection of hydrogen sulfide and methane using a single metal oxide sensor with temperature modulation. Similarly, Oliaee et al. [58] added an AuNPs-Fe_2_O_3_ catalytic filter to enhance the selectivity of a Pt/SnO_2_ methane sensor. Fateminia et al. [59] used a Au-promoted Ce-Zr catalytic filter to enhance the selectivity of a Pt/SnO_2_ methane sensor. Other catalytic filters such as Pt-CeO_2_ [60], Au/MO_x_ (M = Zn, Ti) [61], Pt-TiO_2_, Pt-CeO_2_, and Pt-ZrO_2_ [62] have also been reported.

Shaposhnik et al. [63] used regression analysis and principal component analysis for detection of hydrogen sulfide and methane using a single metal oxide sensor with a minimal training set. By modulating the sensor’s temperature, they significantly increased its response for hydrogen sulfide detection compared to constant temperature mode, resulting in sensitivity enhancement by two to four orders of magnitude. The approach allowed qualitative and quantitative analysis of gas mixtures at concentrations different from those in the training set, addressing practical applications.

The response time denotes the duration required for a sensor to register a change in gas concentration, and the recovery time indicates the period taken for the sensor to return to its initial resistance following the removal of the gas. Due to their remarkable electron mobility, both SnO_2_ and ZnO typically exhibit rapid response and recovery times [64]. Conversely, TiO_2_ and WO_3_ tend to possess slower response and recovery times. Regarding stability, ZnO shines due to its exceptional thermal stability, which permits it to maintain consistent performance even at elevated operating temperatures [65]. SnO_2_ also showcases good stability, while TiO_2_ and WO_3_ may experience degradation over time or under strenuous conditions.

Each metal oxide semiconductor possesses a distinct set of advantages and challenges that must be assessed when designing methane sensors. SnO_2_ is an excellent option for applications requiring real-time methane detection due to its high sensitivity and rapid response and recovery times. Nevertheless, its less precise selectivity and necessity for high operating temperatures may restrict its applicability in certain situations [66]. ZnO provides a balanced combination of sensitivity, response time, and thermal stability. Similar to SnO_2_, however, its selectivity could pose problems, and it typically necessitates high operating temperatures. The capacity of TiO_2_ to function at lower temperatures can be advantageous in contexts where high temperatures are either unfeasible or undesirable. Yet, its reduced sensitivity and slower response and recovery times may curtail its effectiveness for real-time detection. WO_3_, which has been widely used for ozone detection due to its superb selectivity, is an appealing choice for methane detection. However, its slower response and recovery times, along with potential stability issues, could restrict its utility under certain conditions.

## 4. Recent Developments in Metal Oxide Semiconductors for Methane Sensing

Recent years have seen an abundance of innovative strategies designed to enhance the performance characteristics of metal oxide semiconductors for methane detection. These advancements encompass material modifications, nanostructuring, the introduction of hybrid materials, and more [67,68,69,70,71,72]. Each strategy seeks to improve sensitivity, selectivity, response and recovery times, and stability, thereby expanding the capabilities of chemiresistive sensors in methane detection.

**Figure 5:**
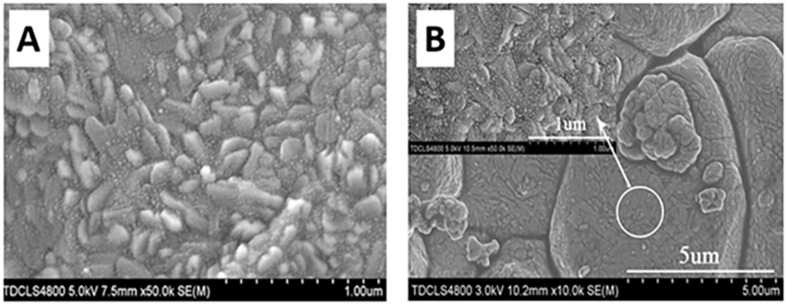
SEM images of (**A**) Pt/VOx and (**B**) Au/Vox films. Reproduced with permission from Refs. [83,84].

Material modifications, such as doping and functionalization, have become increasingly prevalent strategies in the realm of gas sensor development. Doping involves introducing a small amount of an impurity element into the semiconductor to change its properties. For instance, noble metal doping (e.g., Pt, Pd, or Au) into SnO_2_ or ZnO has been reported to enhance their methane-sensing performance due to the creation of additional adsorption sites or alteration of the charge transfer mechanism. Pd stands as the most ubiquitously employed oxidation catalyst for SnO_2_ films in methane gas detection [73]. A myriad of researchers has utilized a variety of techniques to incorporate Pt into SnO_2_, including the sol–gel process, chemical synthesis, and radio frequency sputtering, among others [74,75,76,77,78,79,80]. Fedorenko et al. [81] developed a highly sensitive gas sensor for methane detection based on nanosized Pd-containing tin dioxide obtained through a sol–gel technique. The results showed that the Pd/SnO_2_ nanomaterials retained a cassiterite-type structure. The addition of palladium helped stabilize the particle sizes, resulting in smaller particles compared to the undoped SnO_2_. The sensors based on 1.41% Pd/SnO_2_ exhibited the highest response to methane, with a response ratio (R_0_/R_g_) of 12.4, which exceeded many other reported sensors. These sensors also demonstrated fast response and recovery times (t 0.9  =  6 s and τ_rel0_._1 _ =  10 s) and were capable of detecting methane concentrations in a wide range (47–937 ppm). Utilizing the RF sputtering technology, Haridas and Gupta [82] investigated the impact of different catalyst thicknesses on the sensor’s response. Among the catalysts tested, the SnO_2_-Pd cluster structure exhibited the best performance, achieving a high response of 97.2% at an operating temperature of 220 °C. Notably, a critical thickness of 8–10 nm for Pd clusters facilitated enhanced catalytic activity for adsorbed oxygen and improved the spill-over mechanism, leading to an observed increase in sensor resistance at moderate temperatures (160–220 °C). The maximum response (99.2%) was achieved when the Pd cluster thickness was 10 nm, showcasing its optimal catalytic behavior for methane detection even at a low operating temperature of 160 °C. Liang et al. [83] developed room temperature methane sensors using Pt-decorated VO_x_ (Pt/VO_x_) thin films. The preparation involved sputtering vanadium metal on sapphire substrates, depositing PtNPs, and annealing the films at different temperatures. The Pt/VO_x_ films exhibited irregular rod-shaped particles with an average size of around 100 nanometers (Figure 5A). The Pt/VO_x_ sensors demonstrated good repeatability and sensitivity toward methane at room temperature, with the best performance observed for the sample annealed at 460 °C. This sensor showed a large response value of 18.2 at a methane concentration of 500 ppm. The optimal operating concentration for methane detection was found to be 1500 ppm. They also investigated the gas-sensing properties of Au-decorated VO_x_ thin films (Figure 5B) for methane detection at room temperature [84]. The films were prepared using magnetron sputtering and rapid thermal annealing. The results showed that the Au/VO_x_ films exhibited excellent gas-sensing performance at room temperature, with the highest response observed at a methane concentration of 1500 ppm. The films annealed at 480 °C showed the best response due to their porous and intense grain density. The introduction of AuNPs on the VO_x_ films facilitated a nanometal-semiconductor junction, promoting the dissociation of methane and accelerating the reaction with absorbed oxygen species. Similarly, the introduction of AuNPs has also been conducted to SnO_2_ [85].

Introducing noble metals, typically Pt or Pd, onto the surface of semiconductor metal oxides can undoubtedly enhance the performance of gas sensors. Such promotional effect is primarily attributable to the catalytic activity of noble metals in the oxidation of hydrocarbons. The PdNPs doped within the semiconductor metal oxide materials have the capability to activate the dissociation of oxygen molecules. At elevated temperatures, oxygen molecules weakly bind with the catalyst metal atoms, Pd, resulting in the formation of complexes. These subsequently dissociate into oxygen atoms, which then undergo a spillover process, diffusing onto the surface of the metal oxide. These oxygen atoms finally morph into negatively charged surface ions by gaining electrons from the metal oxide surface, which escalates the quantity of adsorbed oxygen and the molecular ionization conversion rate [86,87]. Upon exposure of the sensor to methane, Pd atoms adsorb hydrogen atoms, facilitating the dissociation of the C−H bond in methane and, thus, lowering the activation energy required for the sensing reaction. The noble metal surface generates hydrogen (H) or methyl (CH_3_) entities, which spillover onto the metal oxide surface, and react with the chemically adsorbed oxygen ions on the sensitive film surface to produce water and free electrons, leading to the formation of weakly bonded Pd^δ+^(CH_4_)^δ−^. This process allows the electrons to return to SnO_2_, leading to a significant reduction in the height of the potential barrier at the Pd/SnO_2_ interface, an increase in electrical conductivity, and a decrease in resistance.

First-principle calculations based on DFT were performed to investigate the effects of Pt doping on the gas-sensing properties of SnO_2_ [88]. The calculations showed that O_2_ molecules can be stably adsorbed on the surface of both undoped and Pt-doped SnO_2_ (110) crystal planes. However, the adsorption energy for O_2_ was found to be lower on the Pt-doped surface compared to the undoped surface. This indicates that O_2_ molecules are more easily adsorbed on the Pt-doped SnO_2_ surface, which may be due to the role of Pt dopants. The calculations evaluated the formation energy of Pt doping at different sites on the SnO_2_ (110) surface. It was found that Pt atoms preferentially substitute the top layer Sn_6_C atoms, as this configuration exhibited the lowest formation energy among the four possible doping sites. The adsorption energy of methane molecules on various adsorption sites of the undoped and Pt-doped SnO_2_ (110) surfaces was calculated. The results showed that the adsorption energy of methane molecules decreased on certain adsorption sites after Pt doping. Specifically, the adsorption energy of the H_3_CH (three H atoms on SnO_2_ surface) adsorption model at the O_p_ (oxygen bridge) adsorption site decreased the most after doping with Pt. The calculations of the total density of states revealed that a new electron state appeared around the Fermi level for the Pt-doped SnO_2_ surface. This new electronic state was mainly attributed to the presence of Pt_5d_ states.

The use of pure Pd metal is associated with certain limitations, including the formation of blisters due to an irreversible transition from the alpha phase of palladium to the beta hydride phase under low hydrogen concentrations at a temperature of 300 K [89]. To mitigate these issues, palladium is often alloyed with a secondary metal (typically 13% to 30% Ag), thereby enhancing its efficacy for detecting hydrogen or hydrocarbons. The Pd-Ag alloy possesses several additional benefits that enhance its appeal for gas sensors, as previously reported [90]. Firstly, the rate of hydride formation in the Pd-Ag alloy is notably lower than that in pure Pd. Moreover, the hydrogen solubility in the alloy actually exceeds that of pure Pd up to an Ag concentration of around 30%, without the silver atoms hindering hydrogen diffusion. Alloys with higher Ag concentrations (up to 45%) have been found to demonstrate increased hydrogen adsorption rates within the temperature range of 30 to 100 °C. The energy barrier for OH formation is also elevated in the presence of a Pd-Ag alloy. Finally, the mechanical properties of polycrystalline Pd-Ag alloys surpass those of pure Pd.

Similarly, noble metal doping also can be used for other types of metal oxide semiconductors. For example, Ghosh et al. [91] developed a methane gas sensor using a ZnO thin film coated with a Pd-Ag alloy. The sensor demonstrated high selectivity and sensitivity toward methane gas at an operating temperature of 100 °C. The sensing mechanism involved the adsorption of oxygen molecules on the ZnO surface, leading to the formation of O_2_^−^ ions. When methane gas was introduced, it reacted with the O_2_ ions, producing H_2_O_2_ as a byproduct. The instability of H_2_O_2_ at the operating temperature caused it to break down into water and oxygen, resulting in a significant change in the sensor’s resistance, allowing for methane detection. Among different Pd-Ag patterns tested, the Pd-Ag dotted structure showed the best performance with an 80% response selectively toward methane and low responses to other gases. The sensor exhibited stable and repeatable performance during a five-day experiment and showed reliable selectivity over a wide range of methane concentrations. In addition, other element doping has also been carried out for enhancing sensing performance, such as Fe-doped SnO_2_ [92]_,_ Os-doped SnO_2_ [93], Sb/Pd-doped SnO_2_ [94], PdPt-doped SnO_2_ [95], Al-doped NiO [96], Pd-doped ZnO [97], Au-doped ZnO [98], Ni-doped In_2_O_3_ [99].

**Figure 6:**
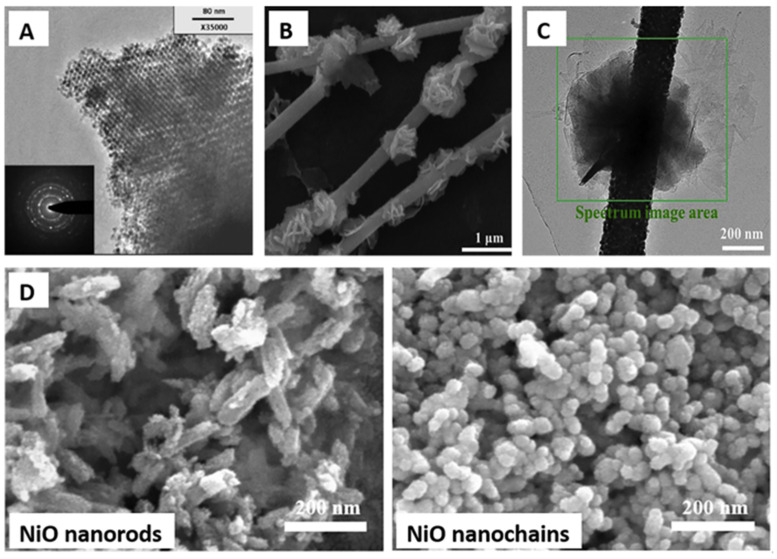
(**A**) TEM image of mesoporous SnO_2_. (**B**) TEM image of flower-like MoS_2_ with porous SnO_2_ nanofibers for methane sensing. (**C**) High-magnification images of SnO_2_/MoS_2_ nanocomposites. (**D**) SEM images of NiO porous nanorods and coral-like nanochains. Reproduced with permission from Refs. [44,100,101].

The advent of nanotechnology has brought a significant paradigm shift in the design of chemiresistive sensors. Researchers have explored various nanostructured metal oxides, such as nanoparticles, nanowires, nanotubes, and nanoplates, for methane sensing. The rationale behind this approach is that nanostructuring can dramatically increase the surface-to-volume ratio of the material, thereby providing more active sites for gas adsorption and enhancing sensitivity. Waitz et al. [44] synthesized mesoporous SnO_2_ (Figure 6A) methane gas sensors using nanocasting from ordered mesoporous KIT-6 silica. The nanocasted SnO_2_ materials exhibited exceptional thermal stability, retaining their structural integrity up to 600 °C with only a minor decrease in specific surface area at 800 °C. In contrast, conventionally prepared SnO_2_ materials experienced a significant loss of porosity during thermal treatment. Wang et al. [100] synthesized hierarchical composites of MoS_2_ nanoflowers anchored on SnO_2_ nanofibers (Figure 6B) for methane gas sensing. The unique nanostructure, combining the flower-like MoS_2_ with porous SnO_2_ nanofibers, resulted in improved gas-sensing properties compared to pure MoS_2_. The SnO_2_/MoS_2_ nanocomposites exhibited a lower optimal operating temperature and higher gas response, reaching a response of 2.014 to 100 ppm methane at an operating temperature of 180 °C. The excellent performance was attributed to the larger surface areas and effective active sites for gas adsorption and desorption provided by the composite structure. Additionally, the presence of n-n junctions between SnO_2_ and MoS_2_ facilitated electron transfer, further enhancing gas sensitivity. Zhang et al. [101] investigated the structural evolution of NiO from porous nanorods (Figure 6C) to coral-like nanochains and its impact on methane-sensing performance. NiO porous nanorods and coral-like nanochains were prepared through annealing a NiC_2_O_4_ precursor at different temperatures and used as sensing materials for methane detection. The coral-like nanochains showed a larger specific surface area compared to porous nanorods. Despite the lower surface area, the NiO coral-like nanochains exhibited remarkably enhanced methane-sensing properties with a response value ranging from 12.8% to 55.8% for methane concentrations from 500 to 4000 ppm at an operating temperature of 320 °C. In contrast, the response of NiO porous nanorods ranged from 4.7% to 26.9% under the same conditions. The improved methane-sensing performance was attributed to the structural evolution from nanorods to nanochains, which influenced the resistivity of the materials and facilitated methane transport. The results suggest that NiO coral-like nanochains hold great potential for high-performance methane gas sensors.

**Figure 7:**
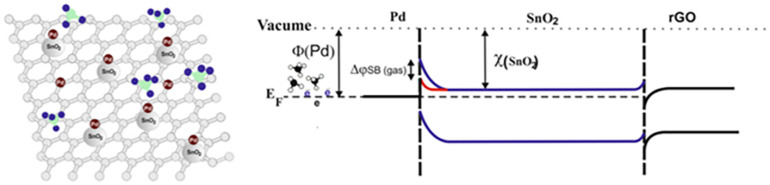
The proposed mechanism for methane sensing using Pd-doped SnO_2_/rGO. Reproduced with permission from Ref. [103].

The development of hybrid materials, which combine two or more distinct types of materials, represents another cutting-edge approach in sensor technology. One common strategy is creating metal oxide–carbon hybrids, where metal oxide nanoparticles are incorporated into carbon materials like CNT or graphene. These hybrids leverage the high conductivity and large surface area of carbon materials, along with the gas-sensing properties of metal oxides, to achieve enhanced sensor performance. For example, Chimowa et al. [102] investigated the gas-sensing properties of vanadium-filled multi-walled carbon nanotubes (V-MWNTs) and compared them with unfilled MWNTs and pure V_2_O_5_. The results revealed that the V-MWNTs showed improved gas-sensing performance, particularly for methane gas detection. The vanadium filling process led to weak physisorption interactions with gases, reducing gas response and recovery times, making the material more suitable for practical gas-sensing applications. The sensitivity of V-MWNTs to methane gas increased by at least 1% compared to unfilled MWNTs. The material exhibited fast response times of 16 s and recovery times of 120 s. The modified Langmuir model with three different adsorption sites provided a good fit to the experimental data, suggesting the existence of multiple adsorption sites with different energies. Nasresfahani et al. [103] developed a room-temperature methane gas sensor using Pd-doped SnO_2_ nanoparticles integrated with reduced graphene oxide (rGO) and partially rGO (PRGO) matrices. The methane gas-sensing properties of the nanocomposites were investigated, and it was found that the Pd-doped SnO_2_/rGO sensor exhibited higher sensitivity (up to 9.5%) for 12,000 ppm methane at room temperature and a quicker response time compared to the Pd-doped SnO_2_/PRGO sensor. The sensing mechanism of the Pd-doped SnO_2_/rGO nanocomposite methane gas sensor involves several key steps (Figure 7). Firstly, the SnO_2_ nanoparticles act as active sites for the adsorption of oxygen molecules from the surrounding air, creating an electron depletion region and increasing the sensor’s resistance. Secondly, the PdNPs in the composite facilitate the dissociation of oxygen molecules, leading to the formation of oxygen ions on the surface of SnO_2_. These PdNPs also serve as excellent adsorption centers for hydrogen atoms, which aids in the dissociation of C-H bonds in methane. When methane gas is present, it reacts with the oxygen ions on the surface of the sensing film, forming weakly bound complexes of Pd(CH_4_). As a result, the electrons return to the SnO_2_, reducing the potential barrier height at the Pd/SnO_2_ interface and increasing the sensor’s conductivity. Additionally, the rGO nanosheets provide a conducting network that facilitates rapid electron transfer, contributing to the sensor’s fast response time. The overall combination of Pd-doped SnO_2_ and rGO results in an efficient and sensitive methane gas sensor at room temperature. Similar work has been reported by Navazani et al. [104,105] and Kooti et al. [106].

Hybridization of different metal oxides is also a strategy. Aifan et al. [107] prepared nanocomposites of SnO_2_-In_2_O_3_ incorporating TiO_2_ for methane gas sensing and catalytic oxidation. The researchers optimized the composition and preparation parameters, finding that the addition of 40 mol% In_2_O_3_ and 20 mol% TiO_2_ resulted in the best-performing nanocomposite. Calcination at 600 °C provided the ideal crystallinity for maximum gas response and catalytic activity. The sensor response increased with higher operating temperatures, peaking at a specific temperature and then decreasing at higher temperatures due to surface interaction with the gas. They revealed that the nanocomposites’ improved performance was attributed to smaller crystallite size, favorable gas adsorption and reaction, and electronic interactions among the components. Overall, these optimized nanocomposites showed promising gas-sensing capabilities, with the highest response reaching 47.2 for methane at an operating temperature of 300 °C. Additionally, the catalytic activity for methane oxidation was significantly enhanced, with a T10% (light-off temperature) of 462 °C and T90% (full combustion temperature) of 597 °C for the nanocomposite containing 20% TiO_2_. Aleksanyan [108] reported a study on a methane sensor based on a three-component nanostructure of SnO_2_/In_2_O_3_/TiO_2_. Two sets of samples with different concentrations of component materials were prepared and thin films were deposited using magnetron sputtering. The nanostructure composed of 70%SnO_2_–10%In_2_O_3_–20%TiO_2_(anatase) showed the highest sensitivity to methane at a working temperature of 350 °C. The sensor demonstrated an almost instant response to 1% methane injection, with the resistance decreasing by nearly five times. The recovery time was about 7 min. Importantly, the sensor was found to be practically insensitive to other gases at this temperature. Chakraborty et al. [109] developed thick-film methane sensors using nanosized SnO_2_ powder containing Sb_2_O_3_ and Pd. The powder was prepared using sonication-assisted simultaneous precipitation, resulting in sensors with optimal resistance and excellent sensitivity to methane. The impedance plots of sensors made with sonication-assisted precipitation matched well with those of high-quality imported Figaro sensors, indicating their comparable performance. The combinations also can be found in NiO-In_2_O_3_ [110], NiO-SnO_2_ [111], Ni_2_O_3_-SnO_2_ [112], SnO_2_-WO_3_ [113,114], β-Ga_2_O_3_-ZnO [115,116], ZnO/ZnO_2_ [117]. Other materials have also been reported for incorporation with metal oxide semiconductors, such as HZSM-5 zeolite/Pd-SnO_2_ [118] and Co_3_O_4_/MoS_2_ [119].

Each of these advancements brings about unique enhancements to the performance of metal oxide semiconductors in methane detection. These experimental results highlight the significant strides that have been made in recent years toward enhancing the performance of metal oxide semiconductors for methane sensing. As the field continues to advance, more innovative strategies and materials are likely to emerge, further improving the capabilities of chemiresistive sensors.

## 5. Sensor Fabrication Techniques and Integration

The fabrication of chemiresistive sensors, specifically those employing metal oxide semiconductors for methane detection, involves a variety of sophisticated techniques. These methods play a critical role in determining the ultimate performance of the sensors. In addition, the integration of these sensors into systems represents another vital aspect that is shaping the landscape of methane detection.

**Figure 8:**
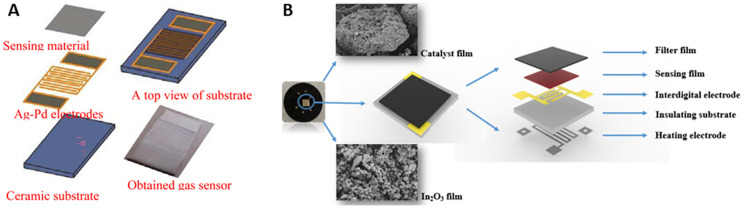
(**A**) Schematic micrograph of the planar ZnO-based gas sensor and obtained gas sensor. (**B**) Schematic drawing and photograph of the sensor devices fabricated by Pd-In_2_O_3_. Reproduced with permission from Refs. [45,121].

Thin-film deposition is extensively used in the fabrication of chemiresistive sensors due to its ability to produce uniform and high-quality films of metal oxides. In the context of methane sensors, thin-film deposition can be used to create a thin, continuous layer of metal oxide on a substrate, which serves as the active sensing element. For example, Gagaoudakis et al. [96] used RF sputtering for Al-doped NiO thin-film deposition. RF sputtering is a physical vapor deposition (PVD) technique that is commonly used to deposit thin films of various materials onto substrates. The process involves bombarding a Ni-Al composite target with high-energy ions, typically from a plasma, which leads to the ejection of target atoms. These ejected atoms then condense on the substrate, forming a thin film. The optimized process parameters, such as the amount of oxygen in the plasma and the doping level of aluminum, resulted in films with over 50% transparency in the visible region and an optical energy band gap of 3.65 eV. Sol–gel method also can be used for thin-film deposition. The sol–gel process is a wet chemical method for preparing oxide materials in the form of thin films, fibers, or powders. It involves the conversion of a sol (a colloidal suspension of nanoparticles) into a gel and subsequent drying and annealing steps to obtain the desired oxide material. For example, Bhattacharyya et al. [120] prepared a solution containing Zn(CH_3_COO)_2_·2H_2_O and isopropanol. Diethanolamine was added to this solution to yield a clear, transparent, and homogeneous sol. The sol contains ZnO nanoparticles in a liquid form. The ZnO sol was spin-coated onto SiO_2_-coated p-Si substrates. The substrate is then rapidly spun, causing the sol to spread uniformly and form a thin film. After spin coating, the samples were heated at 110 °C for 10 min to evaporate the solvent and remove organic residuals, leaving behind the ZnO NPs on the substrate. The samples were then annealed at 600 °C. Annealing is a high-temperature treatment that promotes the crystallization and growth of the ZnO NPs, resulting in the formation of nanocrystalline ZnO thin films. The sol–gel method allows for the precise control of film thickness, composition, and morphology. It is relatively simple, cost-effective, and compatible with various substrate materials, making it suitable for producing thin films for gas-sensing applications. Thin-film deposition techniques can produce high-quality, uniform layers of metal oxide, ensuring good reproducibility of sensor responses. However, the sensor sensitivity may be limited due to the lower surface area-to-volume ratio compared to nanoparticle-based sensors. Screen printing is a cost-effective and scalable technique for fabricating gas sensors. It involves the printing of a paste containing metal oxide particles onto a substrate using a mesh screen. After printing, the substrate is typically subjected to a high-temperature sintering process to form a porous metal oxide layer. The ease of fabrication and potential for mass production make screen printing an attractive method for sensor fabrication. For example, Zhou et al. [45] successfully synthesized flower-like ZnO nanorods, net-like ZnO nanofibers, and ZnO nanobulks using a surfactant-assisted hydrothermal method. They investigated the gas-sensing properties of these nanostructures by fabricating gas sensors using a screen-printing technique on a flat ceramic substrate (Figure 8A). Zhao et al. [121] coated a Pd-In_2_O_3_ film with a Pt-Al_2_O_3_ catalyst film and observed significant improvements in sensor performance (Figure 8B). The screen-printing method used for the catalyst film deposition showed promising results for achieving selective and sensitive methane gas detection, making it a valuable advancement in gas sensor technology. The presence of the catalyst film increased the sensor’s sensitivity to methane by thermally activating the methane molecules during diffusion. Screen-printed sensors can offer high sensitivity due to their porous structure, providing numerous sites for gas adsorption. Nevertheless, the performance can be influenced by the quality of the printed layer and the sintering conditions.

The choice of fabrication method can significantly influence the performance characteristics of the chemiresistive sensors. Beyond fabrication techniques, the integration of chemiresistive sensors into systems facing real-world scenarios is an important aspect that can significantly extend their utility. In addition, the appropriate signal collection and processing technology is also the key to improving the sensor detection performance.

**Figure 9:**
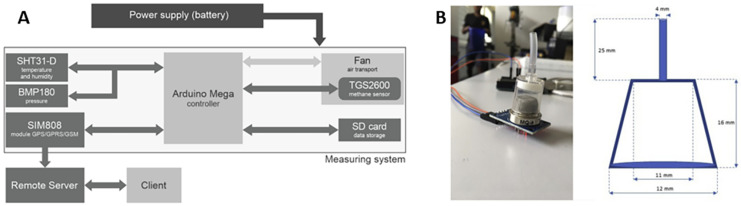
(**A**) System architecture for methane sensing based on the Arduino platform using the TGS2600 sensor. (**B**) The partially closed capsule for direct gas measurement and its dimensions. Reproduced with permission from Refs. [123,125].

Sensor systems can provide real-time monitoring of methane levels over large areas, which is particularly useful for detecting leaks in natural gas infrastructure or monitoring methane emissions from agricultural and industrial sources [122]. The sensors can be wirelessly connected to a central server, where the data are processed and analyzed. Furst et al. [123] focused on the development and evaluation of a portable and low-cost methane measurement system (Figure 9A) based on the Arduino platform using the TGS2600 sensor. This innovative system was applied in two distinct environments: a small ruminant barn and a wastewater treatment plant. Despite some limitations, such as the sensor’s low repeatability and cross-sensitivity to certain gases, the system showed promising results. It successfully captured the variations in methane concentrations inside the barn, correlated with animal presence and ventilation conditions. Additionally, the system demonstrated good response to methane concentrations below 50 ppm at the wastewater treatment plant, with low sensitivity to interfering gases like ammonia. To further enhance the system’s performance, the study recommended regular calibrations and the incorporation of a ventilation system to improve the sensor’s response.

Recently, Butturini and Fonollosa [124] reported a low-cost metal oxide semiconductor sensor system for measuring methane concentrations in aquatic ecosystems. The experimental setup included a flow-through configuration with a peristaltic pump controlling the sample flow rate. The sensor’s response was found to be stable and sensitive to methane concentrations in the range of 0.1 to 4.5 μM at water temperatures between 18 °C and 30 °C. A calibration model was built using data from different days and temperatures, providing a prediction error of 0.085 μM. However, the system showed cross-sensitivity to sulfide interference, and correction for sulfide concentration was necessary in samples with low methane content and high sulfide levels. Overall, the low-cost metal oxide semiconductor sensor system proved to be a valuable tool for rapid and affordable methane measurements in aquatic ecosystems, enabling potential applications in online monitoring and continuous multiple measurements. Fakra et al. [125] developed a low-cost gas measurement device using MQ-4 and MQ-8 sensors to monitor methane and hydrogen concentrations in biomass gasification systems. Three measurement techniques were compared: the first involved injecting gas into an airtight chamber, providing good repeatability but limited linearity; the second required direct injection in an open environment, resulting in improved linearity but decreased repeatability due to external influences; the third technique used direct injection into a partially closed capsule (Figure 9B), yielding the best results with high linearity (R^2^ = 0.9973 for methane and 0.9472 for hydrogen) and improved repeatability (maximum standard deviation of 14.26% for methane and 17.73% for hydrogen). The partially closed capsule technique allowed for linear measurements up to 20% methane and 13.33% hydrogen concentrations, making it a promising, low-cost solution for monitoring gasification systems in remote areas.

A sensor system has been developed for specific evaluation of the concentrations of hydrogen and methane emitted by termites using a semiconductor gas sensor [126]. They conducted experiments with different termite species, numbers of termites, and temperature conditions. The results showed that both hydrogen and methane concentrations increased with an increase in the number of termites, and the presence of a wood specimen further enhanced gas emissions. The highest gas concentrations were observed at 35 °C, indicating active feeding behavior. Among the termite species, *Zootermopsis nevadensis*, a dampwood termite, emitted the highest levels of hydrogen and methane. Importantly, the gas detection system demonstrated its potential as a nondestructive method for termite detection. The ability to detect termite emissions of hydrogen and methane could offer an effective and accurate means of early termite attack detection in wood, providing an alternative to visual inspection and supporting chemical-free termite management strategies. Dobrzyniewski et al. [127] reported a Principal Component Regression as the calibration method for a gas sensor array system for monitoring the dry methane reforming process. The results showed that the sensor array successfully determined the quantitative parameters of the reforming process, including Inlet Molar Ratio (IMR), Outlet Molar Ratio (OMR), and Methane Conversion Level (MCL). The average error for IMR determination was about 7% in the range of 0.6–1.5, while for OMR and MCL, the errors were below 1%. This demonstrated the effectiveness of the sensor array system in accurately monitoring the process.

Machine learning is a subfield of artificial intelligence that focuses on the development of algorithms and statistical models that enable computers to learn and improve their performance on a specific task without being explicitly programmed. The key idea is to allow machines to learn from data, identify patterns, and make decisions based on that acquired knowledge. In a paper published by Barriault et al. [128], machine learning is used for gas detection and quantification using a single metal oxide semiconductor sensor embedded in a microchannel. The sensor generates time–response data when exposed to different gas mixtures. Machine learning is employed in two main aspects: (1) A large number of features are extracted from the raw sensor data, representing various characteristics of the gas responses. Machine learning algorithms are utilized to select the most relevant features for the classification and regression tasks. The selected features are used to represent the data in a more compact and informative manner, enhancing the performance of the models. (2) For gas classification, machine learning models are trained to distinguish between pure methane, pure ethane, and binary mixtures of the two gases. The models learn from the labeled data and then can accurately classify unknown gas samples. For regression, the models predict the concentrations of methane and ethane in gas mixtures. This allows for quantification of gas mixtures, even when dealing with low concentrations of ethane. For classification, the best accuracy achieved on the test set was 100% using the k-NN model for both pure methane and 3% ethane mixtures. In regression, the MLP model provided the most accurate predictions for 3% ethane, with only a 10.9% error for methane and a 14.9% error for ethane concentrations.

## 6. Challenges and Limitations

While the potential of metal oxide semiconductors in chemiresistive methane sensors is evident from the advances made in their development and deployment, there are still several limitations and challenges that need to be addressed to further enhance their effectiveness and reliability in practical applications.

Selectivity: Many metal oxides like SnO_2_ and ZnO respond to other reducing gases besides methane, making selective detection of methane difficult. Strategies to improve selectivity may include surface functionalization or incorporation of selective filters/membranes.

Humidity: Methane sensors can be affected by humidity and water vapor. Some metal oxides are more prone to humidity interference than others. Approaches to mitigate humidity effects include sensor encapsulation, hydrophobic coatings, and data processing algorithms.

Operating Temperature: Elevated temperatures (>200 °C) are typically required for optimal methane response. This increases power consumption and can shorten sensor lifetime. New materials that operate at lower temperatures while maintaining methane sensitivity are needed.

Detection Limits: Current detection limits for many metal oxide methane sensors are in the hundreds of ppm range, whereas some applications require lower ppb detection. Improving sensitivity to detect trace methane levels remains a key challenge.

Durability: Long-term stability and durability of metal oxide sensors in real-world methane detection applications need improvement. Exposure to harsh environments, contaminants, and temperature/humidity fluctuations can degrade performance over time.

Response/Recovery Times: Response times to methane exposure are generally fast for metal oxides (<1 min). However, recovery times are typically slower, limiting the ability to track rapid methane concentration changes. Faster baseline recovery is desirable.

Interfering Gases: Methane detection can be affected by gases commonly present in environmental samples, like CO_2_, VOCs, NO_x_, etc. Improved selectivity in complex gas mixtures and environmental conditions is needed.

Advanced Material Science: Solutions for enhancing sensor selectivity, reducing operating temperature, and improving long-term stability may be found in advanced material science. For example, developing novel metal oxide materials with unique catalytic properties or structural modifications such as doping or hybridization can enhance selectivity and reduce operating temperatures. Nanoengineering techniques, such as constructing nanostructured surfaces or interfaces, can also offer promising routes to enhance sensitivity and selectivity. There is a need for continuous exploration of new materials and material combinations that could offer improved sensing performance. Research into advanced material modification techniques, such as doping, alloying, or constructing heterostructures, could provide pathways to overcome existing limitations.

Design Improvements: Sensor design modifications, such as the development of integrated heating elements, can provide precise temperature control to enhance sensor performance and longevity. Additionally, designing sensor structures that allow for effective heat dissipation can help reduce the impact of high operating temperatures on sensor stability and lifespan.

Data Processing and Machine Learning: Sophisticated data processing algorithms and machine learning techniques can be employed to improve sensor performance, especially in terms of selectivity and stability. These techniques can help differentiate between the responses to different gases and correct for baseline drift and other long-term changes in sensor behavior. The integration of advanced computational techniques like machine learning and AI with sensor technology is expected to be transformative. Machine learning algorithms can be utilized to process sensor data, differentiate between various interfering gases, and mitigate the effects of environmental fluctuations, thereby enhancing the reliability and accuracy of methane detection.

Green Manufacturing: As environmental consciousness increases globally, the trend toward green and sustainable manufacturing practices will likely influence sensor fabrication. Researchers may focus on developing environmentally friendly synthesis methods and utilizing renewable or less hazardous materials.

Through ongoing research and innovation in these areas, the goal of developing reliable, efficient, and cost-effective chemiresistive methane sensors using metal oxide semiconductors can be realized, leading to significant societal and environmental benefits.

## 7. Conclusions

The application of semiconductor metal oxide materials in chemiresistive methane gas sensors has undergone remarkable advancements, making significant strides in sensitivity, selectivity, and sensor fabrication techniques. Noble metal doping, nanostructuring, and hybrid materials have substantially enhanced the sensitivity and selectivity of these sensors, providing promising results for practical methane detection. Furthermore, innovative sensor fabrication methods, such as thin-film deposition and screen printing, offer cost-effective and scalable approaches for sensor production. However, several challenges and limitations persist in the field of metal oxide-based methane sensors. Achieving high selectivity remains a significant challenge, as the sensors can be influenced by interfering gases present in the environment, leading to false positives. Additionally, the requirement for high operating temperatures to facilitate gas-sensing reactions complicates sensor design and increases energy consumption. To address these challenges, advanced material science techniques have been explored to develop novel metal oxide materials with improved properties, aiming to enhance sensitivity and reduce operating temperatures. Design improvements, such as integrated heating elements for precise temperature control, have also been investigated to enhance sensor stability and longevity. Moreover, the integration of machine learning and data processing algorithms has shown promising results in improving selectivity and mitigating baseline drift. The recent developments in semiconductor metal oxide-based chemiresistive methane gas sensors hold great promise for practical applications. The improvements in sensitivity, selectivity, and stability achieved through material innovations and design modifications open up new opportunities for efficient methane monitoring and leak prevention. However, ongoing research is essential to overcome the remaining challenges and fully unlock the potential of these sensors. Green manufacturing practices should also be considered to align with environmental sustainability goals. Overall, the advancements in this field present a positive outlook for reliable methane detection, contributing to environmental protection and safety.

## Figures and Tables

**Table 1:** Example industrial methane sensors and typical detection range.

Sensor Model	Manufacturer	Typical Detection Range
TGS2611	Figaro	500~10,000 ppm
MQ-4	Zhengzhou Winsen	300~10,000 ppm
MPS005	Nevada Nano Methane Gas Sensor	500~1500 ppm
Cubic SJH-100	CO2Meter	0~100% LEL
RS-CH4-*-2	Renke	0~100% LEL

* LEL: Lower explosive limit.

**Table 2:** Comparison of different methane sensors.

Sensor Types	Mechanisms	Advantages	Disadvantages
Optical sensors	Detect changes in light waves due to interaction of analyte with receptor.	Non-destructive; Immune to electromagnetic interference; Can operate without oxygen.	High cost; High power consumption; Lack of significance and distinctiveness of methane optical absorption region.
Calorimetric sensors	Measure heat produced from chemical reaction and correlate to reactant concentration.	Simplistic design; Portable; Easy to manufacture; Easy to manufacture; Good selectivity; Can operate in harsh environments.	Low detection accuracy; Susceptible to cracking, catalyst poisoning, and oversaturation; High power consumption; short lifespan.
Pyroelectric sensors	Convert thermal energy into electrical energy based on pyroelectricity.	Non-destructive; Can operate without oxygen; Good sensitivity and responsivity; Wide measuring range; Operate at room temperature.	High cost; High power consumption; Immobile; Difficult to manufacture.
Chemiresistive sensors	Absorption of gas on the surface of metal oxide surface changes its conductivity, which is measured to determine gas concentration.	Low cost; Lightweight and robust; Long lifespan; Resistant to poisoning.	Poor selectivity; Small and high operational temperature range; Slow recovery rate; Significant additive dependency; Affected by temperature, humidity, degradation; Sensitive to humidity.
Electrochemical sensors	Measure target gas concentration by oxidizing/reducing the gas at an electrode and measuring current.	Low cost; Non-hazardous materials; High boiling points and low volatility; Good selectivity; Can detect small leaks. Solid-state-based: No leakage; Safe; Robust; Good selectivity for methane.	Amperometric-based: susceptible to leakage and evaporation; Hazardous materials; Slow response time. Ionic liquid-based: Susceptible to leakage; Slow response time. Solid-state-based: Requires high temperature; Unable to detect low gas concentrations; Susceptible to degradation or loss of electrolyte.
